# The Role of Interleukin-6 (IL-6) in the Systemic Inflammatory Response in Xenograft Recipients and in Pig Kidney Xenograft Failure

**DOI:** 10.3389/fimmu.2021.788949

**Published:** 2021-12-08

**Authors:** Guoqiang Zhang, Hayato Iwase, Qi Li, Takayuki Yamamoto, Abhijit Jagdale, Mohamed B. Ezzelarab, David Ayares, David K. C. Cooper, Hidetaka Hara, Gangcheng Wang

**Affiliations:** ^1^ Department of General Surgery, Affiliated Cancer Hospital of Zhengzhou University, Henan Cancer Hospital, Zhengzhou, China; ^2^ Xenotransplantation Program, Department of Surgery, University of Alabama at Birmingham, Birmingham, AL, United States; ^3^ Department of Surgery, Thomas E. Starzl Transplantation Institute, University of Pittsburgh, Pittsburgh, PA, United States; ^4^ Revivicor Inc., Blacksburg, VA, United States; ^5^ Center for Transplantation Sciences, Department of Surgery, Massachusetts General Hospital, Boston, MA, United States

**Keywords:** baboon, po-inflammatory cytokines, inflammation, IL-6, kidney, pig, serum amyloid A, xenotransplantation

## Abstract

**Background:**

In pig-to-baboon transplantation models, there is increasing evidence of systemic inflammation in xenograft recipients (SIXR) associated with pig xenograft failure. We evaluated the relationship between systemic inflammatory factors and pig kidney xenograft failure.

**Methods:**

Baboons received kidney transplants from genetically engineered pigs (n=9), and received an anti-CD40mAb-based (n=4) or conventional (n=5) immunosuppressive regimen. The pig kidney grafts were monitored by measurements of serum creatinine, serum amyloid A (SAA), white blood cell (WBC) and platelet counts, plasma fibrinogen, and pro-inflammatory cytokines (baboon and pig IL-6, TNF-α, IL-1β).

**Results:**

Six baboons were euthanized or died from rejection, and 3 were euthanized for infection. Changes in serum creatinine correlated with those of SAA (r=0.56, p<0.01). Serum *baboon* IL-6 was increased significantly on day 1 after transplantation and at euthanasia (both p<0.05) and correlated with serum creatinine and SAA (r=0.59, p<0.001, r=0.58, p<0.01; respectively). but no difference was observed between rejection and infection. Levels of serum *pig* IL-6, TNF-α, IL-1β were also significantly increased on day 1 and at euthanasia, and serum pig IL-6 and IL-1β correlated with serum creatinine and SAA. The level of serum baboon IL-6 correlated with the expression of IL-6 and amyloid A in the baboon liver (r=0.93, p<0.01, r=0.79, p<0.05; respectively).

**Conclusion:**

Early upregulation of SAA and serum IL-6 may indicate the development of rejection or infection, and are associated with impaired kidney graft function. Detection and prevention of systemic inflammation may be required to prevent pig kidney xenograft failure after xenotransplantation.

## Introduction

Organ transplantation is the preferred method of treatment of end-stage organ failure, but a severe shortage of deceased human donor organs is a major limitation. Great progress has been made in the study of xenotransplantation since the first chimpanzee-to-human kidney xenotransplant was performed by Reemtsma in 1963. The pig has since been identified as a potential source of organs for clinical transplantation (1), but there remain several barriers in pig-to-primate organ xenotransplantation that have to be overcome. One of these is the systemic inflammatory response in the xenograft recipient (SIXR) to the presence of a pig organ (2–4).

Inflammation is a complex biological defensive response to infection or biological stress, in which pro-inflammatory cytokines activate the immune system. However, an excessive inflammatory response can result in various pathological states, such as coagulation and/or immune dysregulation (5). In kidney allotransplantation, inflammatory signals promote T cell activation and play an important role in rejection, impairing T cell tolerance and preventing long-term graft survival (6). In pig organ xenotransplantation, SIXR, as evidenced by a sustained rise in C-reactive protein (C-RP), precedes the development of a consumptive coagulopathy (7).

Kidney graft failure is largely documented by an increase in the serum creatinine, with confirmation by histopathologic assessment of kidney graft biopsies. We suggest that SIXR may be closely associated with pig kidney graft failure, and therefore the detection and prevention of an inflammatory response may play an important role in prolonging the survival of a pig xenograft in a nonhuman primate (NHP). C-RP is an acute phase protein that is synthesized by hepatocytes in response to proinflammatory cytokines, in particular interleukin-6 (IL-6) and is widely used to evaluate an inflammatory response. However, it can be significantly inhibited by IL-6 receptor blockade (e.g., with tocilizumab) (8–10). Serum amyloid A (SAA) is a more sensitive and convenient marker than C-RP when anti-inflammatory therapy is being administered (11–13). Other inflammatory markers, e.g., serum histones, free triiodothyronine, plasma fibrinogen, and platelet count, can help monitor the inflammatory response or health status of NHP recipients of pig xenografts (14, 15).

The pro-inflammatory cytokines, e.g., IL-6, TNF-α, and IL-1β, involved in the pathological processes of rejection and infection in baboon xenograft recipients (3, 4, 16, 17), and may be associated with coagulation dysfunction (7, 14). IL-6 increases to a high level in baboons after pig kidney xenotransplantation. *In vitro* studies, baboon IL-6 can activate pig aortic endothelial cells (18). However the roles of pro-inflammatory cytokines in SIXR-associated rejection or infection remain unclear.

We measured inflammatory factors in NHP recipients of pig kidney grafts, and provide evidence that SIXR is associated with pig kidney graft failure from rejection or with infection.

## Materials and Methods

### Animals and Kidney Transplantation

Baboons (n=9) (Oklahoma University Health Sciences Center, Oklahoma City, OK) received a life-supporting kidney graft from genetically-engineered pigs (Revivicor, Blacksburg, VA) (**Table 1**). The operative procedures and management of the baboons have been detailed previously (19, 20).

**Table 1:** Pig genetic engineering, immunosuppressive therapy, recipient survival, and causes of death with tocilizumab treatment.

Baboon Number	Donor pig genetic engineered phenotype	Maintenance IS therapy	Survival (days)	Cause of Death
**Group A (Rejection)**				
B14115	GTKO/CD46/TBM/EPCR/CD47/HO1	Conventional therapy	1	HAR
B10815	GTKO/CD46/TBM/EPCR/CD47/HO1	Anti-CD40mAb-based	90	AHXR
B14214	GTKO/CD46/hvWF	Conventional therapy	32	AHXR
B5415	GTKO/CD46/TBM/EPCR/CD47/HO1	Conventional therapy	15	AHXR
B9015	GTKO/CD46/TBM/EPCR/CD47/HO1	Conventional therapy	13	AHXR
B15815	GTKO/CD46/TBM/EPCR/CD47/HO1	Conventional therapy	12	AHXR
**Group B (Infection)**				
B9313	GTKO/CD46/CD55/TBM/EPCR/CD39	Anti-CD40mAb-based	136	infection
B17315	GTKO/CD46/CD55/EPCR/TFPI/CD47	Anti-CD40mAb-based	237	infection
B17615	GTKO/CD46/CD55/EPCR/TFPI/CD47	Anti-CD40mAb-based	260	infection

HAR, hyperacute rejection; AHXR, acute humoral xenograft rejection; GTKO, α1,3-galactosyltransfearse gene-knockout; TBM, thrombomodulin; EPCR, endothelial protein C receptor; HO1, heme oxygenase-1; hvWF, human von Willebrand factor; TFPI, tissue factor pathway inhibitor. Conventional therapy consisted of only FDA-approved agents (various combinations of CTLA4-Ig, tacrolimus, rapamycin, mycophenolate mofetil).

All animal care was in accordance with the “Principles of Laboratory Animal Care” formulated by the National Society for Medical Research and the “Guide for the Care and Use of Laboratory Animals” prepared by the Institute of Laboratory Animal Resources and published by the National Institutes of Health (NIH publication No. 86-23, revised 1985). All procedures were approved by the Institutional Animal Care and Use Committees of the University of Pittsburgh and the University of Alabama at Birmingham.

### Immunosuppressive and Anti-Inflammatory Therapy

All baboons received thymoglobulin (ATG) and anti-CD20mAb (Rituximab) as induction therapy. Maintenance immunosuppressive regimens are summarized in **Table 1** (together with other information, e.g., graft survival and cause of death). The immunosuppressive regimens were either anti-CD40mAb-based or conventional therapy-based (which consisted of only FDA-approved agents – with various combinations of CTLA4-Ig, tacrolimus, rapamycin, mycophenolate mofetil) (21–23). Anti-CD40mAb (2C10R4, a chimeric rhesus IgG4) was provided by the NIH NHP Reagent Resource (24). Anti-inflammatory therapy was administered in an attempt to reduce inflammation. All baboons received interleukin-6 receptor (IL-6R) blockade with tocilizumab (10mg/kg on days −1, 7, 14, and thereafter every 2 weeks; Actemra, Genentech, South San Francisco, CA), and the TNF-αantagonist, etanercept (0.5mg/kg on days 0, 3, 7, and 10, Amgen, Thousand Oaks, CA).

### Monitoring of Recipient Baboons

Whole blood and serum samples were obtained from recipients before and serially after transplantation for measurement of blood cell counts, chemistry (kidney function, etc.), coagulation parameters, and the inflammatory response (based on measurement of SAA and selected serum cytokines). Blood cell counts were measured by standard methods (Central Laboratory of Presbyterian Hospital, Pittsburgh, PA).

#### Serum Amyloid A

SAA was measured by the Rapid Test for Inflammation & Infection kit (Accuplex, Maynooth, Co Kildare, Ireland), as an indication of inflammation, per the manufacturer’s instructions (13). The results of the SAA assay were categorized into groups, using the following scoring system (1=no or minimal inflammation; 2=moderate inflammation; 3=severe inflammation).

#### Serum Cytokine Assays

Serum cytokine assays were performed as previously described (18). The levels of cytokines (baboon IL-6, TNF-α, IL-1β) in baboon serum were measured by cytometric bead array (CBA) with a human Inflammatory Cytokine Kit (No. 551811; BD Biosciences, San Jose, CA), according to the manufacturer’s instructions. LSR II flow cytometry (BD Biosciences) was used to collect data, which were analyzed using CBA analysis software (BD Biosciences). Levels of pig IL-6 in baboon serum were measured by ELISA using a porcine IL-6 Quantikine ELISA Kit (No. P6000B; R&D Systems, Minneapolis, MN), following the manufacturer’s instructions. Optical density was measured by using a Wallac Victor3 1420 Multilabel Counter (Perkin Elmer, Waltham, MA) at 450nm, with the correction wavelength set at 540nm or 570nm.

### Immunohistochemistry

Baboon liver tissues were obtained at euthanasia, immediately fixed in formalin, and embedded in paraffin. Sections (4μM) were cut. IL-6 and amyloid A expression were evaluated in 7 baboon livers by immunohistochemistry (IHC) (13). After xylene, they were rehydrated, endogenous peroxidase activity was eliminated, and antigen was retrieved. The samples were incubated with the primary and secondary antibodies, followed by diaminobenzidine staining, and examined with an optical microscope (Olympus Optical, Tokyo, Japan). For staining, the primary antibodies were rabbit polyclonal IL-6 antibody (ab6672, Abcam, Cambridge, UK), and rat anti-amyloid A (Accuplex, Maynooth, Co Kildare, Ireland) and the secondary antibodies were goat anti-rat and goat anti-rabbit (Ab#97057, Cat#Ab6721, Abcam, Cambridge, MA). Quantitative analysis of expression was determined by measurement of the mean optical density (MOD) using Image J software (National Institute of Mental Health, Bethesda, MA). Five visual fields of each sample were randomly selected, and 5 areas were randomly selected from each visual field. The mean optical density was calculated finally.

### Statistical Analysis

Statistical analyses were performed with GraphPad Prism 7 software (GraphPad Software, La Jolla, CA). Continuous variables were expressed as mean +/- SD. The unpaired Welch`s t test or Mann-Whitney U-test for variables (such as serum creatinine, WBC, platelet, fiboringen, SAA score, inflammatory factors). The Spearman rank correlation respectively analyzed correlations between serum creatinine and other variables, between SAA and inflammatory factors (such as baboon or pigIL-6), between serum baboon IL-6 and IL-6 or amyloid A in baboon livers. Differences were considered to be statistically significant at p<0.05.

## Results

### Clinical Course

Based on the clinical course and graft histopathology, the baboons were divided into 2 groups - Rejection group (n=6) and Infection group (n=3). In the Rejection group, 5 baboons received conventional immunosuppressive therapy, and only one received an anti-CD40mAb-based regimen, whereas all 3 baboons in the Infection group received the anti-CD40 mAb-based regimen. In the Rejection group, one pig kidney underwent hyperacute rejection, and 5 underwent acute humoral xenograft rejection. In the Infection group, all 3 baboons were euthanized for systemic infection (at 136, 237, and 260 days) with functioning grafts (**Table 1**).

### Serum Creatinine

**Figure 1:**
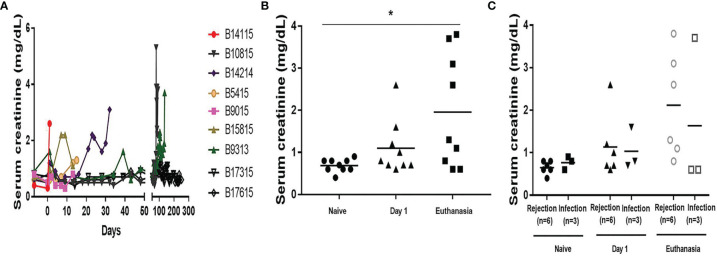
Serum creatinine in baboons with pig kidney grafts. **(A)** Levels of serum creatinine in the baboons (n=9) at different time-points. **(B)** The difference of the levels of serum creatinine in the baboons (n=9) at pre-Tx (naïve), day 1 after operation, and at euthanasia. **(C)** The differences in serum creatinine between the rejection (n=6) and infection (n=3) groups at pre-Tx (naïve), day 1 after operation, and at euthanasia. (*p < 0.05).

An increase in serum creatinine was the most important indicator of failing pig kidney function after xenotransplantation (**Figure 1A**). Serum creatinine rose in the days before euthanasia became necessary (euthanasia vs pre-Tx, 1.96+/-0.45 vs 0.69+/-0.05mg/dL, p<0.05) (**Figure 1B**), but there was no significant difference between the Rejection and Infection groups on day 1 or at euthanasia (day 1, 1.13+/-0.31 vs 1.03+/-0.28mg/dl; at euthanasia, 2.12+/-0.50 vs 1.63+/-1.03mg/dL) (**Figure 1C**), although in the Infection group the serum creatinine was significantly increased in only one baboon (B9313) at euthanasia.

### Systemic Inflammatory Response

**Figure 2:**
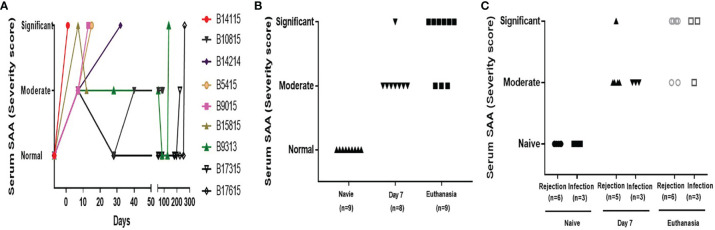
Serum amyloid A in baboons with pig kidney grafts. **(A)** SAA scores in the baboons (n=9) at different time-points. **(B)** The difference in SAA scores in the baboons (n=9) at pre-Tx (naïve), day 7 after operation, and at euthanasia. **(C)** The difference in SAA scores between the rejection (n=6 or 5) and infection (n=3) groups at pre-Tx (naïve), day 7 after operation, and at euthanasia. The severity of the systemic inflammatory response was based on the results of the SAA assay, using a simple scoring system (1 = no inflammation; 2 = moderate inflammation; 3 = severe inflammation).

A systemic inflammatory response after transplantation was detected by increases in SAA levels, especially at the time of euthanasia (**Figures 2A, B**). In the Rejection group, B14115 died from hyperacute rejection on day 1, when the SAA was significantly increased (**Figure 2A**). In the remaining 5 baboons, SAA levels were significantly increased in 3, and moderately increased in 2 (**Figure 2C**). In the Infection group, SAA was increased during the first week and significantly or moderately increased at euthanasia (**Figure 2C**). In the absence of rejection or infection, the SAA remained negative or moderate.

### White Blood Cell (WBC) and Platelet Counts and Plasma Fibrinogen

**Figure 3:**
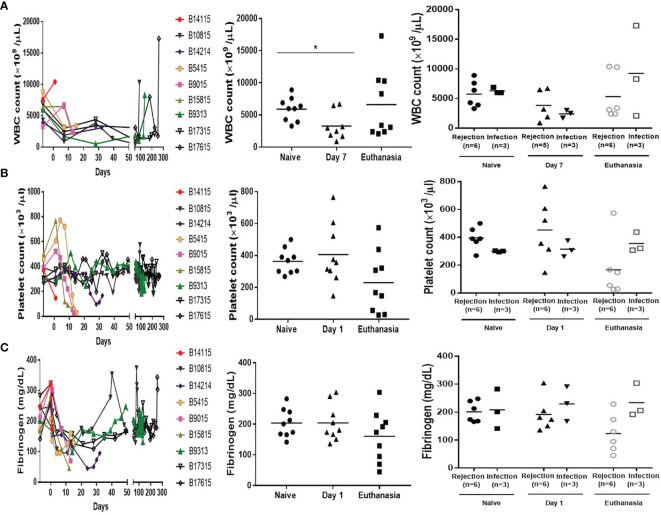
White blood cell and platelet counts, and plasma fibrinogen levels in baboons with pig kidney grafts. **(A)** WBC counts in the baboons (n=9) at different time-points (Left); The difference in the WBC count in the baboons (n=9) (Middle), and between the rejection (n=6 or 5) and infection (n=3) groups at pre-Tx (naïve), day 7 after operation, and at euthanasia (Right). **(B)** Platelet counts in the baboons (n=9) at different time-points (Left); The difference in the platelet count in the baboons (n=9) (Middle), and between the rejection (n=6) and infection (n=3) groups at pre-Tx (naïve), day 1 after operation, and at euthanasia (Right). **(C)** Plasma fibrinogen in the baboons (n=9) at different time-points (Left); The difference in the plasma fibrinogen in the baboons (n=9) (Middle), and between the rejection (n=6) and infection (n=3) groups at pre-Tx (naïve), day 1 after operation, and at euthanasia (Right). (*p < 0.05).

The WBC count was significantly decreased following induction therapy, and remained low (**Figure 3A**, left and middle). On post-transplant day 7, there was no statistical difference between the groups (3840+/-1185 vs 2400+/-379/µL) (**Figure 3A**, right). At euthanasia, the mean WBC count in the Infection group was higher than in the Rejection group, but with no significant difference (9233+/-4413 vs 5333+/-1595/µL) (**Figure 3A**, right).

Platelet counts remained within the normal range in both groups on day 1 (452+/-91 vs 315+/-33/µl, p>0.05) (**Figure 3B**, right). At euthanasia, the mean platelet count in the Rejection group was lower than in the Infection group (167+/-85 vs 356+/-41/µl), but was not significantly different (p=0.09) (**Figure 3B**, right).

Changes in plasma fibrinogen were similar to those in platelet count (**Figure 3C**, left and middle). It was maintained within the normal range at day 1 after operation in both groups (230+/-26 vs 192+/-24mg/dl, p>0.05) (**Figure 3C**, right). At euthanasia, it was lower in the Rejection group than in the Infection group (234+/-35 vs 124+/-28/µl) but was not significantly different (p=0.06), especially in 2 baboons (B9015, B15815) (**Figure 3C**, right).

### Serum Cytokines

**Figure 4:**
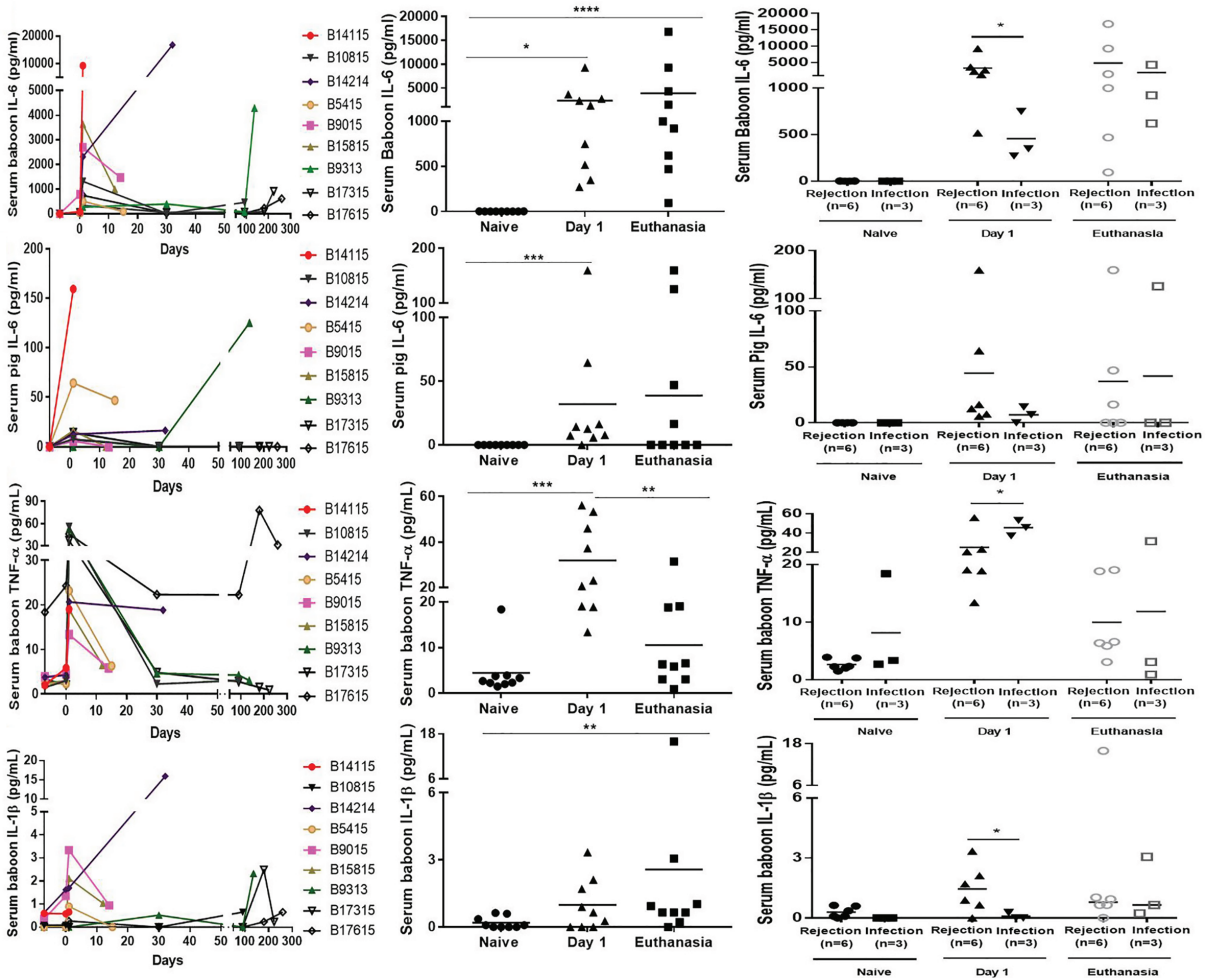
Levels of serum cytokines in baboons with pig kidney graft. **(A)** Levels of serum baboon IL-6 in the baboons (n=9) at different time-points (Left); The difference of serum baboon IL-6 in the baboons (n=9) (Middle), and between the rejection (n=6) and infection (n=3) groups at pre-Tx (naïve), day 1 after operation, and at euthanasia (Right). **(B)** Levels of serum pig IL-6 in the baboons (n=9) at different time-points (Left); The difference of serum pig IL-6 in the baboons (n=9) (Middle), and between the rejection (n=6) and infection (n=3) groups at pre-Tx (naïve), day 1 after operation, and at euthanasia (Right). **(C)** Levels of serum TNF-α in the baboons (n=9) at different time-points (Left); The difference of serum TNF-α in the baboons (n=9) (Middle), and between the rejection (n=6) and infection (n=3) groups at pre-Tx (naïve), day 1 after operation, and at euthanasia (Right). **(D)** Levels of serum IL-1β in the baboons (n=9) at different time-points (Left); The difference of serum IL-1β in the baboons (n=9) (Middle), and between the rejection (n=6) and infection (n=3) groups at pre-Tx (naïve), day 1 after operation, and at euthanasia (Right). (*p < 0.05, **p < 0.01, ***p < 0.001, ****p < 0.0001).

Serum *baboon* IL-6 was significantly increased on day 1 and at euthanasia (**Figure 4A**, left and middle). In the Rejection group, the levels of serum baboon IL-6 were increased on day 1, particularly in B14115 that died from hyperacute rejection (9260pg/mL); in the remaining 5 the levels of serum baboon IL-6 ranged from 516 to 3636pg/mL. The levels of serum *baboon* IL-6 were also significantly increased (range from 272 to 748pg/mL) in the Infection group, but were higher in the Rejection group (3291+/-1273 vs 456+/-148pg/mL, p<0.05) (**Figure 4A**, right). At euthanasia, the levels were high in both groups, and not statistically different Rejection group 4847+/-2769 vs Infection group 1943+/-1178pg/mL) (**Figure 4A**, right).

Serum *pig* IL-6, produced by the pig kidney graft, was detectable in some baboons in both groups on day 1 and at euthanasia, especially in B14115 (159pg/mL) and B9313 (125pg/mL) (**Figure 4B**, left and middle). Although higher in the Rejection group than in the Infection group on day 1 (44+/-25 vs 7+/-4pg/mL), and lower than in the Infection group at euthanasia (37+/-26 vs 42+/-42 pg/mL), there was no significant difference on day 1 or at euthanasia (**Figure 4B**, right).

Serum TNF-α was significantly elevated on day 1, and then recovered or was elevated at euthanasia in both groups (**Figure 4C**, left and middle). It was lower in the Rejection group than in the Infection group on day 1 (25+/-6 vs 46+/-5pg/mL, p<0.05). The levels of serum TNF-α in 4 baboons (B10815, B9313, B17315, B17615) with long survival were very high on day 1 (range 37-53pg/mL), but there was no difference between the two groups at euthanasia (10+/-3 vs 12+/-10pg/mL, p>0.05) (**Figure 4C**, right).

The levels of serum IL-1β were low throughout the course of the experiments (**Figure 4D**, left and middle), The detection rate and levels of serum IL-1β were higher in the Rejection group than in the Infection group on day 1, In only one baboon (B17615) was a low level of serum IL-1β detected in the Infection group. There was no statistica0y difference between the two groups at euthanasia (3+/-3 vs 1+/-1pg/mL, p >0.05) (**Figure 4D**, right).

### Correlation of Serum Creatinine With SAA and Serum Cytokines

**Figure 5:**
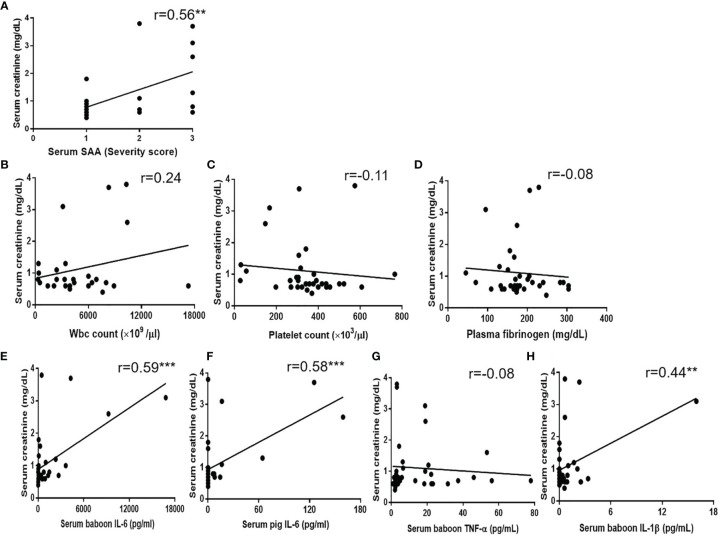
Correlations between serum creatinine with **(A)** SAA score, **(B)** WBC count, **(C)** platelet count, **(D)** plasma fibrinogen, **(E)** serum baboon IL-6, **(F)** serum pig IL-6, **(G)** serum TNF-α, **(H)** serum IL-1β. Significant positive correlations were found between serum creatinine and SAA score (**p < 0.01), serum baboon IL-6 (***p < 0.001), serum pig IL-6 (***p < 0.001) or serum IL-1β (**p < 0.01).

Significant positive correlations were found between serum creatinine and SAA (r=0.56, p<0.01) (**Figure 5A**). Serum baboon IL-6, serum pig IL-6, and serum IL-1β (but *not* serum TNF-α) also positively correlated with serum creatinine (r=0.59, p<0.001; r=0.58, p<0.001; r=0.44, p<0.01, respectively) (**Figures 5E–H**), but serum creatinine did not correlate with WBC or platelet counts, or plasma fibrinogen (**Figures 5B–D**).

### Correlation of SAA With Cytokines (Serum Baboon/Pig IL-6, Serum IL-1β)

**Figure 6:**
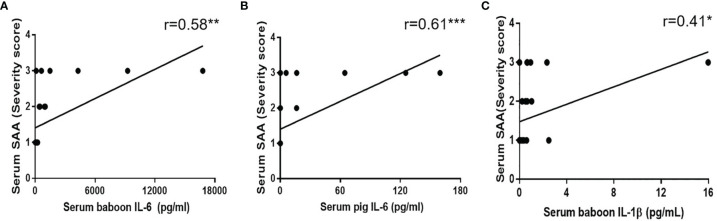
Correlation between SAA score with **(A)** serum baboon IL-6, **(B)** serum pig IL-6, **(C)** serum IL-1β. Significant positive correlations were found between SAA score and serum baboon IL-6 (**p < 0.01), serum pig IL-6 (***p < 0.001) or serum IL-1β (*p < 0.05).

SAA and serum cytokines (serum baboon/pig IL-6, serum IL-1β) positively correlated with serum creatinine. The correlation between the inflammatory response and cytokines was further be analyzed. Significant changes in serum baboon IL-6 correlated with SAA (r = 0.58, p< 0.01) (**Figure 6A**). However, the level and detection rate of serum pig IL-6 was lower than of serum baboon IL-6. Serum pig IL-6 also positively correlated with SAA (r = 0.61, p<0.001) (**Figure 6B**). IL-1β is mostly secreted by monocytes or macrophages, and is involved in inflammatory disease (25, 26). Correlation analysis showed that IL-1β positively correlated with SAA (r=0.41, p<0.05) (**Figure 6C**).

### Serum Baboon IL-6 Level Is Associated With Expression of IL-6 or Amyloid A in the Baboon Livers

**Figure 7:**
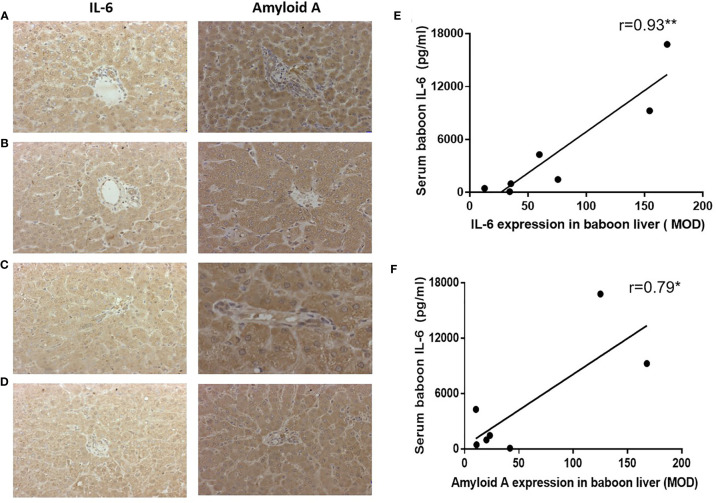
Expression of IL-6 or amyloid A in baboon livers detected by IHC, and correlation with serum baboon IL-6 levels. **(A)** Expression of IL-6 and amyloid A in the liver of a baboon that experienced anaphylactic shock (without transplantation) (positive control), **(B)** Expression of IL-6 and amyloid A in the liver of a baboon with a pig kidney graft that underwent hyperacute rejection (while receiving tocilizumab and etanercept) (positive control). **(C)** Expression of IL-6 and amyloid A in the liver of a representative baboon with a pig kidney graft that was undergoing antibody-mediated rejection (while receiving tocilizumab and etanercept). **(D)** Expression of IL-6 and amyloid A in the liver of a representative baboon with a pig kidney graft that was undergoing serious infection (while receiving tocilizumab and etanercept). **(E, F)** Significant positive correlations were found between serum baboon IL-6 levels and expression of IL-6 (**p < 0.01) or amyloid A (*p < 0.05) in baboon livers.

Expression of IL-6 and amyloid A in the native livers in (i) a baboon that experienced anaphylactic shock after ATG administration (without xenotransplantation or anti-inflammatory therapy) (B11015) (**Figure 7A**), and in (ii) a baboon that hyperacutely rejected a pig kidney graft (B14115) (**Figure 7B**), showed highly-positive staining (and high serum levels of IL-6 and SAA), and were used as positive controls. IL-6 and amyloid A staining in baboon livers (B9015, B9313) represented the *Rejection* (**Figure 7C**) and Infection (**Figure 7D**) groups.

Expression of IL-6 and amyloid A were strongly positive in 7 baboon livers (Rejection group, n=6, and Infection group, n=1). Serum baboon IL-6 positively correlated with IL-6 expression in the baboon livers (r=0.93, p<0.01) (**Figure 7E**), and with amyloid A expression (r=0.79, p<0.05) (**Figure 7F**).

## Discussion

We previously documented evidence of a persistent systemic inflammatory response (SIXR) in immunosuppressed baboons after pig kidney xenotransplantation. SIXR was associated with procoagulant and inflammatory innate immune cells, and possibly with failure of the pig xenograft (3–7).

When a pig organ is exposed to SIXR, thrombocytopenia and a fall in fibrinogen indicate the development of thrombi in the pig graft (7, 23). In the present study, our data showed early dramatic falls in platelet count and plasma fibrinogen before euthanasia that preceded a rise in serum creatinine. This was most obvious in baboons receiving conventional immunosuppressive therapy. Therefore, we suggest that falls in platelet count or plasma fibrinogen may be precursors of graft failure induced by rejection, but are not associated with infection. However, falls in platelet count or plasma fibrinogen did not significantly correlate with the rise in serum creatinine.

Measurement of acute phase proteins, e.g., C-RP, SAA, is known to be valuable in monitoring for a systemic inflammatory response, and these can be promoted by IL-6 (8). IL-6R blockade, e.g., with tocilizumab, has been shown to be beneficial in allo- and possibly xeno-transplantation (27–30). In our previous studies in baboons with pig kidney grafts, treatment with tocilizumab completely prevented a rise in C-RP even when graft failure or SIXR was developing (4, 14). However, the level of SAA was not affected by tocilizumab, and remained significantly increased, and therefore appeared to be a more reliable indicator of SIXR (13). Our present study indicates that the rise in SAA was associated with a rise in serum creatinine, but monitoring of SAA could not distinguish between rejection or infection, as both were associated with a high level of SAA.

IL-6 is a pro-inflammatory cytokine produced by innate immune cells, including monocytes and macrophages, lymphocytes, endothelial cells, and dendritic cells after recognition of the danger signals  (31, 32). In our previous studies, high levels of serum IL-6 were detected in baboon recipients of pig kidney grafts, and were associated with inflammation and coagulation dysregulation (7, 18). In the present study, the level of serum baboon IL-6 in baboons that were euthanized or died from rejection was significantly increased, and was higher than in those euthanized for infection. Serum baboon IL-6 correlated with increases in serum creatinine and SAA, and with expression of amyloid A and IL-6 in the baboon liver. Therefore, serum baboon IL-6 could be a useful indicator for inflammation and impending pig graft failure. Although low, the level of serum pig IL-6 was also positively associated with the increase in SAA and serum creatinine, and may be a supplementary indicator for inflammation and pig graft failure.

TNF-α is another pro-inflammatory cytokine that can regulate the expression of pro-inflammatory-related genes, tissue factor, and leukocyte antigen class I in porcine aortic endothelial cells, and may contribute to activation of complement and procoagulant changes in porcine endothelial cells, with increased tissue factor expression (16, 17, 33, 34). Strategies that block TNF-α may prove useful in xenotransplantation (35, 36). In the present study, a high level of TNF-α was observed on day 1, suggesting that the initial rise of TNF-α was related to the surgical procedure. Etanercept (an anti-TNF-α antibody), beneficial in the treatment of rheumatoid arthritis (37, 38), might be beneficial in baboons undergoing pig organ transplantation if administered on the day of operation or on the previous day. However, our data did not show that the level of TNF-α correlated with the serum creatinine.

Human IL-1β can induce the expression of adhesion molecule genes, chemokines, and tissue factor in pig aortic endothelial cells, and human IL-1β cooperates with TNF-α to induce expression of swine leukocyte antigen (SLA) class-I (16). Pig IL-1β can also activate human umbilical vein endothelial cells (17). It is suggested that IL-1β is likely to promote inflammation and coagulation after pig organ xenotransplantation. However, IL-1β levels did not increase after pig artery patch or heart graft xenotransplantation (39). In the present study, IL-1β was increased at the time of euthanasia, and appeared to be higher during rejection than infection. Changes in IL-1β correlated with those in serum creatinine and SAA, suggesting that IL-1β could be a supplementary indicator of inflammation and impending pig kidney graft failure.

In summary, the present study suggests a relationship between SIXR and pig graft failure. Measurement of serum creatinine, SAA, and pro-inflammatory cytokines (serum baboon and pig IL-6 and IL-1β) in baboon recipients may prove useful in indicating whether a baboon recipient is developing rejection or infection, though it might be insufficient to distinguish conclusively between the two. However, whether increases in pro-inflammatory factors (serum baboon and pig IL-6 and IL-1β) are simply markers of inflammation or are definitively contributing to pig kidney graft failure requires further investigation. Nevertheless, there was a correlation between SAA and serum creatinine. Similarly, serum baboon and pig IL-6 and IL-1β (especially serum IL-6) correlated with SAA and serum creatinine. Therefore, serum baboon and pig IL-6 and SAA may serve as useful biomarkers for inflammation and pig kidney graft failure associated with rejection or infection, and might allow improved monitoring and management of primates with pig organ grafts.

## Data Availability Statement

The original contributions presented in the study are included in the article/supplementary material. Further inquiries can be directed to the corresponding authors.

## Ethics Statement

All procedures were approved by the Institutional Animal Care and Use Committees of the University of Pittsburgh and the University of Alabama at Birmingham.

## Author Contributions

GW and HH designed the research and supervised the experiment. GZ carried out the experiments, analyzed the data, and drafted the article. HI, QL, TY, and AJ contributed to the experiments. DA provided the pigs (from Revivicor, Blacksburg, VA). DC, ME, and HH revised the article. All authors contributed to the article and approved the submitted version.

## Funding

The work at the Universities of Pittsburgh and Alabama at Birmingham was supported in part by NIH grant #U19 AI090959/08. The work at the Zhengzhou University was supported in part by key scientific and technological projects of Henan Province (202102310109), and by key projects jointly constructed by Henan Provincial Health Commission and the Chinese Ministry (SBGJ202002018).

## Conflict of Interest

DA is an employee of Revivicor, Blacksburg, VA.

The remaining authors declare that the research was conducted in the absence of any commercial or financial relationships that could be construed as a potential conflict of interest.

The Handling Editor HG has declared a past co-authorship with one of the authors DC.

## Publisher’s Note

All claims expressed in this article are solely those of the authors and do not necessarily represent those of their affiliated organizations, or those of the publisher, the editors and the reviewers. Any product that may be evaluated in this article, or claim that may be made by its manufacturer, is not guaranteed or endorsed by the publisher.

## Abbreviations

C-RP, C-reactive protein; IL-6, interleukin-6; NHP, nonhuman primate; SAA, serum amyloid A; SIXR, systemic inflammation in xenograft recipients.
